# Prediction Surface Morphology of Nanostructure Fabricated by Nano-Oxidation Technology

**DOI:** 10.3390/ma8125468

**Published:** 2015-12-04

**Authors:** Jen-Ching Huang, Ho Chang, Chin-Guo Kuo, Jeen-Fong Li, Yong-Chin You

**Affiliations:** 1 Department of Mechanical Engineering, Tungnan University, New Taipei City 22202, Taiwan; jc-huang@mail.tnu.edu.tw; 2 Graduate Institute of Manufacturing Technology, National Taipei University of Technology, Taipei 10608, Taiwan; yunchin7@gmail.com; 3 Department of Industrial Education, National Taiwan Normal University, Taipei 10610, Taiwan; chinguo7@yahoo.com.tw (C.-G.K.); j7616@ntnu.edu.tw (J.-F.L.)

**Keywords:** atomic force microscopy (AFM), nano-oxidation, diamond-like carbon (DLC), back propagation neural network (BPN)

## Abstract

Atomic force microscopy (AFM) was used for visualization of a nano-oxidation technique performed on diamond-like carbon (DLC) thin film. Experiments of the nano-oxidation technique of the DLC thin film include those on nano-oxidation points and nano-oxidation lines. The feature sizes of the DLC thin film, including surface morphology, depth, and width, were explored after application of a nano-oxidation technique to the DLC thin film under different process parameters. A databank for process parameters and feature sizes of thin films was then established, and multiple regression analysis (MRA) and a back-propagation neural network (BPN) were used to carry out the algorithm. The algorithmic results are compared with the feature sizes acquired from experiments, thus obtaining a prediction model of the nano-oxidation technique of the DLC thin film. The comparative results show that the prediction accuracy of BPN is superior to that of MRA. When the BPN algorithm is used to predict nano-point machining, the mean absolute percentage errors (MAPE) of depth, left side, and right side are 8.02%, 9.68%, and 7.34%, respectively. When nano-line machining is being predicted, the MAPEs of depth, left side, and right side are 4.96%, 8.09%, and 6.77%, respectively. The obtained data can also be used to predict cross-sectional morphology in the DLC thin film treated with a nano-oxidation process.

## 1. Introduction

As the functional requirements of technological products increase, the dimensions of microsystems have decreased to the nanoscale, and such microsystems have become increasingly integrated and multifunctional. However, as miniaturization of electronic components increases, nanoscale photolithography has encountered a bottleneck due to the limitations of light wavelength and light diffraction masking.

Atomic force microscopy (AFM), which is one of the important representative instruments of scanning probe microscopy (SPM), has unique capabilities because when the distance between AFM and the material surface is several to tens of nanometers, the van der Waals force produced in between can be used as a feedback and measuring mechanism when performing microscopic analysis and measurement of the physical properties on material surface [1,2]. An AFM can be used not only for observing surfaces but also for fabricating nanoscale structures. The AFM technique has provided a simple, but useful, method for nanofabrication. The AFM-based nanolithography techniques developed in the past decades include mechanical scratching [3,4,5,6,7], anodization [8,9,10] and nanoscale electrical discharge machining [11], and nano-scale near-field photolithography [12]. Researchers have also attempted to fabricate nano structures by using the nanometer-scale movement and probe of an AFM as a micro tool. Due to its high resolution, AFM is applicable in scanning probe lithography (SPL). The SPL is a next-generation lithography technique that enables observation of the nanoscale or even atomic-scale surface structures and features, at high-resolution, through the interaction between the AFM probe and the surface. The effectiveness of SPL is comparable to that of lithography, and SPL has great potential for development [6,7,8,9,10,11,12]. However, creating molds at the nano-scale is a limiting factor. The versatility and amenability of the technique make it applicable for generating nanomolds. Tseng studied the scratches made by AFM on nickel thin film, and took the normal force applied by probe during scratching and scratching direction as the parameters of experiment. The experiment revealed a logarithmic relationship between the depth of the scratch and the normal force applied by the probe [13]. Hsu used AFM to make nano-scratches and nanogroove structures on PMMA thin film. After cutting appropriately, an electrode with a 50 nm gap was obtained [14]. Chen covered an ITO substrate with PMMA thin film and used a mechanical lithographic process to fabricate a nanogroove with 150 nm width and 35 nm depth, and also used electrode polymerization to acquire a polypyrrole nanowire with 20 μm length and 350 nm width [15].

The nano-oxidation technique, also called partial oxidation nanolithographic technique, is a novel lithographic technique in which the required oxidized patterns are directly transferred onto silicon chips without the need to go through the complicated optical lithographic process. This technique is operated by the way of local anodizing of the probe in an atmospheric environment to fabricate different kinds of oxidized thin films. An AFM is used to perform the nano-oxidation technique in SPL, which can be performed at a nanoscale resolution [16]. Snow used the nano-oxidation technique to apply negative bias on a H-passivated silicon surface in order to produce an oxidized line with 1–2 nm height and 10–30 nm width [17]. Huang *et al.* [18] used combinations of various oxidized nanoline segments to generate complicated nano-oxide patterns. Nanopatterns fabricated by nano-oxidation obtained complex nanopatterns in the range of 1000 × 1000 nm^2^. Huang *et al.* [19] introduced the concept of using a polygon to produce a circular pattern. The effects of the number of sides in the polygon on the height of oxide circular pattern were explored. It was observed that the oxide height increases with the number of sides of the polygon pattern when the radius is the same. Additionally, the oxide is higher on p-Si (100) than p-Si (111) and n-Si (111). Huang *et al.* [20,21] investigated the surface conditions of silicon wafers with native oxide layers (NOL) or hydrogen-passivated layers (HPL) and their effects on nano-oxidation and wet etching.

Diamond-like-carbon (DLC) is a generic term for a class of materials that can be synthesized by various well-established routes. The resulting phases are diamond-like and have a hardness and mechanical properties comparable to those of crystalline diamond [22]. Since DLC has high hardness, wear resistance, and chemical stability, but a low coefficient of friction, DLC is very suitable as a mold material for nanoscale molds. Diamond-like carbon has increasing industrial uses. Examples include coatings for video tapes and hard drive disks, coating on razor blades and high-temperature electronics, just to name a few. Its tribological properties make it well suited for a variety of applications [23]. Since DLC can be patterned and is inherently durable, this material is an ideal candidate for molding technologies. However, tools can only use a diamond cutter to machine the high hardness diamond-like carbon by traditional hard machining methods, and tool life is not long. Therefore, using AFM-based mechanical scratching nanolithography to manufacture nanostructures on DLC requires a diamond probe. The high cost and short life of the probe make it unsuitable for manufacturing DLC nanostructures. If conductive DLC is used as a machining target to be applied with the oxidation technique, the DLC surface can be transformed to carbon dioxide, which can be dissipated in gas form. Then, a groove structure with nanometer or micrometer scale can be formed on the DLC surface. Kim combined AFM, the nano-oxidation technique, and BPN to establish three models for an etching process. The models had increased accuracy in predicting process information [24]. Khanmohammadi used BPN to predict the average particle size of titanium dioxide by infrared reflectance spectroscopy [25]. Watson used AFM and thenano-oxidation technique to make a patternized design on a DLC, which was then successfully used as a mold to perform rollover [22].

Most studies in which the nano-oxidation technique has been applied in different materials and in which a neural network has been used to predict results stress the effects of a single parameter on the height and width of oxide, but few have discussed the synthetic effects of over two parameters on application of the nano-oxidation technique. No studies in the literature have directly predicted shape (morphology) after processing. The appearance of the fabricated nanostructure by the nano-oxidation technique is a complex three-dimensional surface and, therefore, only if the value of the height or width are known, it cannot understand the shape (morphology) to produce the nanostructures. Therefore, this study uses AFM to carry out visualizable experiment of the nano-oxidation technique, and takes a DLC thin film as the sample. The study explores the voltage applied, oxidation time of probe and probe speed, and establishes a database of nano-oxidation machining. The data are to be analyzed by BPN and MRA. Furthermore, the machining parameters of the nano-oxidation technique are established. The study predicts the depth and width of machining, and acquires a prediction model for the nano-oxidation technique on DLC thin film.

As used herein, the development of prediction models can predict the complete 3D shape after processing, rather than just get the height or width of the data. Therefore, the experimental results of this study are applicable in the DLC film surface machining process, and the complex structure pattern of processing applications. The objective was to provide a highly-reliable DLC nanostructure fabrication technology that the industry can use to produce nanostructure molds.

## 2. Experimental Details

### 2.1. Sample Preparation

The material used by the nano-oxidation technique is DLC thin film, and its substrate is silicon, which was provided by the Kinik Company Ltd. (Taipei, Taiwan). The silicon substrate is coated on a layer of conductive diamond-like carbon film. The DLC wafers were cleaned with DI water (Milli-Q, Millipore, New York, NY, USA) for 10 min, rinsed with isopropyl alcohol (IPA) for 15 min, and dried with air blown at room temperature.

### 2.2. Nano-Oxidation Experiments

**Figure 1:**
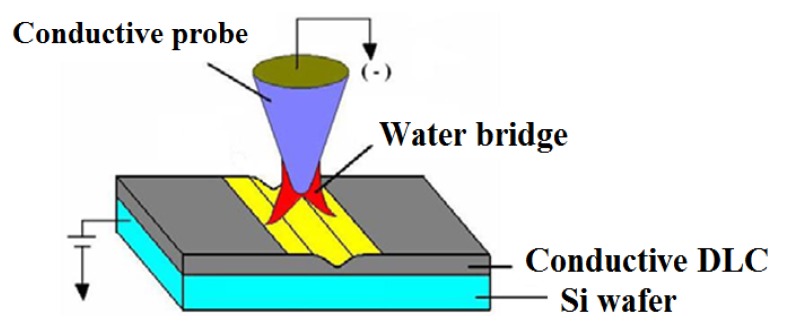
The schematic of nano-oxidation nanolithography on DLC.

An AFM system (Veeco, AFM D3100, New York, NY, USA) equipped with a DCP-20 probe (NT-MDT, Tempe, AZ, USA) was used to perform the nano-oxidation technique on conductive diamond-like carbon thin film by electroluminescent etching. When the conductive probe was 1–2 nm from the DLC wafer surface, a liquid bridge formed between them and produced capillary condensation. Charging the probe with a negative bias voltage and grounding the DLC-coated wafer disassociates the water film on the surface of the DLC piece into hydrogen and hydroxide ions, owing to the strong electric field between the probe surface and DLC. The hydroxide ions and DLC film form a electrochemical reaction, and are converted to carbon dioxide; by means of this method we can manufacture nanoscale grooves. Therefore, an AFM with a conductive probe is used to fabricate micron/nano-scale complex patterns on the surface of DLC thin film by nano-oxidation nanolithography. Figure 1 shows the probe.

The electrochemical reactions between the probe surface and DLC are as follows:

(1) $$\text{In DLC}:\;DLC{\;}_{\left(s\right)}+\;2H_{2}O\;\to \;4H^{+}\;+\;4e^{-}\;+\;{CO}_{2}$$

(2) $$\text{In probe}:\;4H_{2}O\;+\;4e^{-}\;\to \;2H_{2}\;+\;4{OH}^{-}$$

The entire electrochemical reaction is as follows:

(3) $${DLC}_{\left(s\right)}\;+\;2H_{2}O_{\left(l\right)}\;\to \;{CO}_{2(g)}\;+\;2H_{2(g)}$$

All the experiments and measurements were performed at 24 °C and 60% relative humidity. During the nano-oxidation points experiments, the oxidation time for each nanopoint is 1–5 s under an applied voltage of 8–10 V. During the nano-oxidation lines experiments, the length of the nano-oxidation cycle, including the forward and backward lines, was 1 μm under an applied voltage of 8–10 V and a probe speed of 0.1–0.3 μm s^−1^. The probe was moved perpendicular to its major axis. When measuring AFM images, the measuring probe was moved in a direction parallel to that of the probe’s major axis.

## 3. Establishment of Prediction Model

The applied voltage (*v*), the oxidation time (*t*) of the probe, and the speed (*s*) of the probe were taken as independent variables in the MRA and BPN algorithm, and the depth and width (including left width and right width) of oxide as the dependent variables.

The cross section of the groove is similar to the V-shape after the nano-oxidation process. For analysis of the groove width, the V-shaped bottom is the cutoff point, the width of the groove is divided into left and right sides.

Additionally, three independent variables, *v* × *t*, *v* × *v* and *t* × *t*, are increased in order to understand the effects of parameter expansion on the predictive power of regression analysis. Since the applied voltage (*v*) and oxidation time (*t*) have interaction effects on the nano-oxidation process, and its effects are non-linear, the three independent variables, *v* × *t*, *v* × *v* and *t* × *t*, are added to improve the model’s predictive ability of MRA and BNP; this approach has also been found in [9]. Table 1 shows the independent variables used for MRA of point machining. Table 2 shows the settings for independent variables used for MRA of line machining. The voltages (*v*) are set to 8 V, 9 V, and 10 V; the oxidation time (*t*) of probe is 1 s, 2 s, 3 s, 4 s, and 5 s; and the speeds (s) of the probe are 0.1 µm·s^−1^, 0.2 µm s^−1^, and 0.3 µm s^−1^.

**Table 1:** Setting of independent variables of MRA during point machining.

No.	Independent Variable
Database 1	*v*, *t*
Database 2	*v*, *t*, *v* × *t*
Database 3	*v*, *t*, *v* × *v*
Database 4	*v*, *t*, *t* × *t*
Database 5	*v*, *t* , *v* × *t*, *v* × *v*, *t* × *t*

**Table 2:** Setting of independent variables of MRA during line machining.

No.	Independent Variable
Database 1	*v*, *s*
Database 2	*v*, *s*, *v* × *s*
Database 3	*v*, *s*, *v* × *v*
Database 4	*v*, *t*, *s* × *s*
Database 5	*v*, *s*, *v* × *s*, *v* × *v*, *s* × *s*

The settings for independent variables in the BPN algorithm are the same as in the MRA. The BPN model employs a two-layer hidden layer and 2–4 neural nodes together with two learning rates and five independent variables, which results in 10 combinations of variables. The output results of the 10 combinations of variables already set are compared by a trial and error method. The best models are selected to compare with MRA results.

After the nano-oxidation process is performed on the DLC thin film, a nanogroove structure forms on the DLC surface. After measurement of data, the initial machining information is obtained. After that, the measured data is for MRA. Minitab software is used to perform a multiple regression equation. After verification with the experimental data, it is compared with BPN. For BPN, the first step is to enter the experimental values. The selected BPN type is feed-forward back-propagation. The training function is Levenberg–Marquardt; the input conversion function is a hyperbolic tangent conversion function; the output conversion function is a linear conversion function; the number of hidden output layers is five; the number of input variables is five; the learning rate is 0.001–0.005; and the number of iterations is 10,000. The above parameters are used to perform learning and training. The trained index values are to simulate prediction operations. The prediction results are compared with those of the MRA. Finally, the data acquired from BPN are used in Matlab to construct a 3D diagram of the prediction model.

## 4. Results and Discussion

### 4.1. Nano-Oxidation Points

The effects of different levels of applied voltages and oxidation times were compared by testing nano-oxidation at point patterns on a DLC wafer; the experiments and measurements were performed at 24 °C and 60% relative humidity. During the nano-oxidation points experiments, the oxidation time for each nanopoint was 1–5 s under applied voltages of 8–10 V.

**Figure 2:**
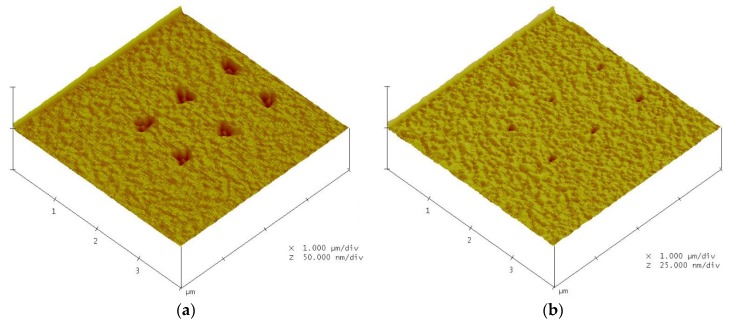
The AFM images for nano-oxidation with oxidation time of 5 s and applied voltages of (**a**) 10 V and (**b**) 8 V.

**Figure 3:**
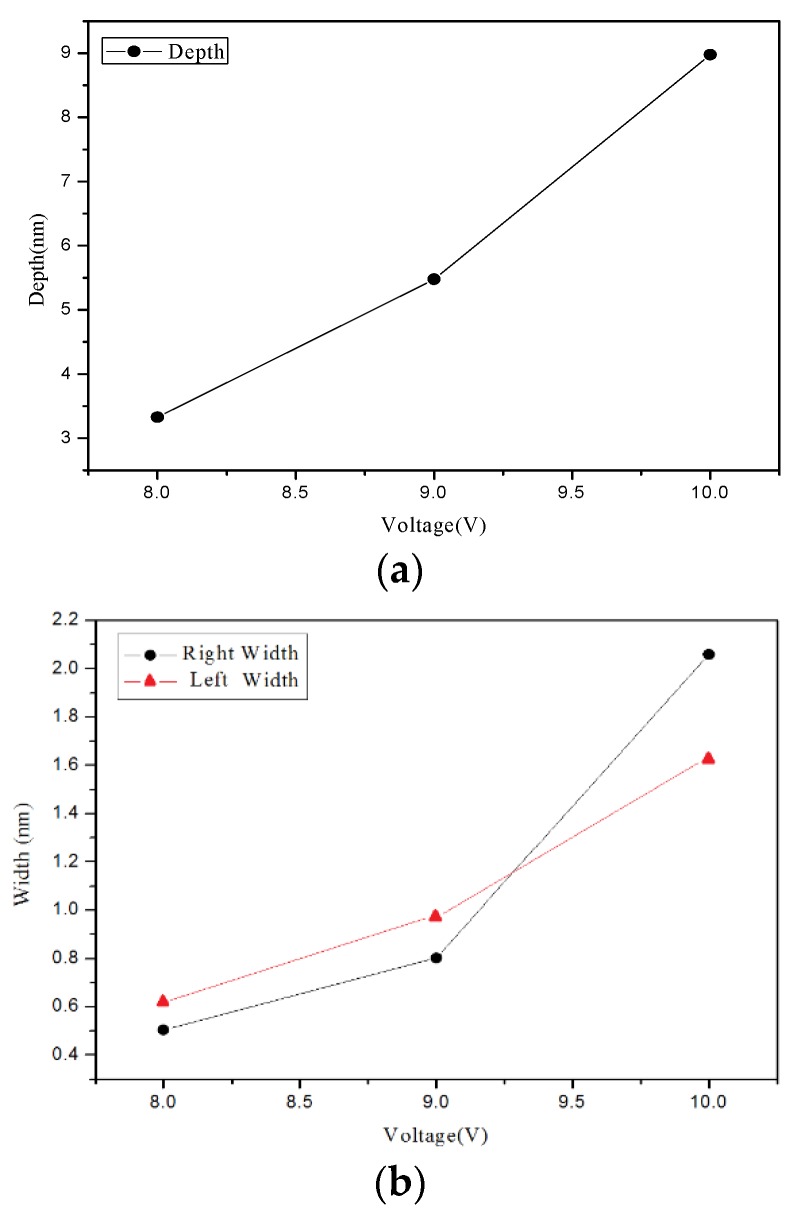
Chart of relationship between point groove depth and width at different applied voltages: (**a**) depth, and (**b**) right width and left width.

**Figure 4:**
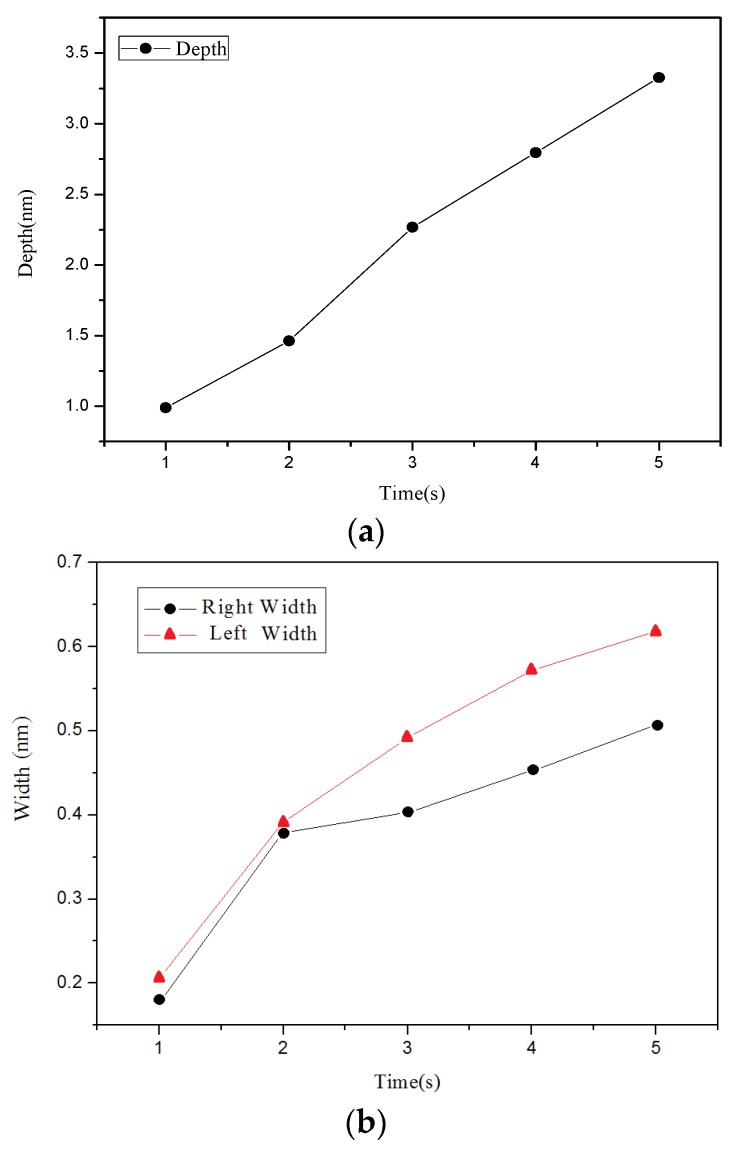
Relationship chart between the point groove depths and widths in different oxidation time: (**a**) depth, and (**b**) right width and left width.

Figure 2 present the AFM measurement results. Changes in the grooves depth and width due to the different applied voltages are shown in Figure 3. The changes in the grooves’ depth and width due to the different oxidation time are shown in Figure 4. Figure 2, Figure 3 and Figure 4 show that the grooves depth and width increased proportionally with applied voltage and oxidation time.

### 4.2. Nano-Oxidation Lines

The effects of different applied voltages and probe speeds were analyzed by testing nano-oxidation at line patterns with a length of 1000 nm on a DLC wafer, using a probe speed of 0.1–0.3 μm·s^−1^ at 8–10 V in an environment with a relative humidity of 60% and temperature of 24 °C.

**Figure 5:**
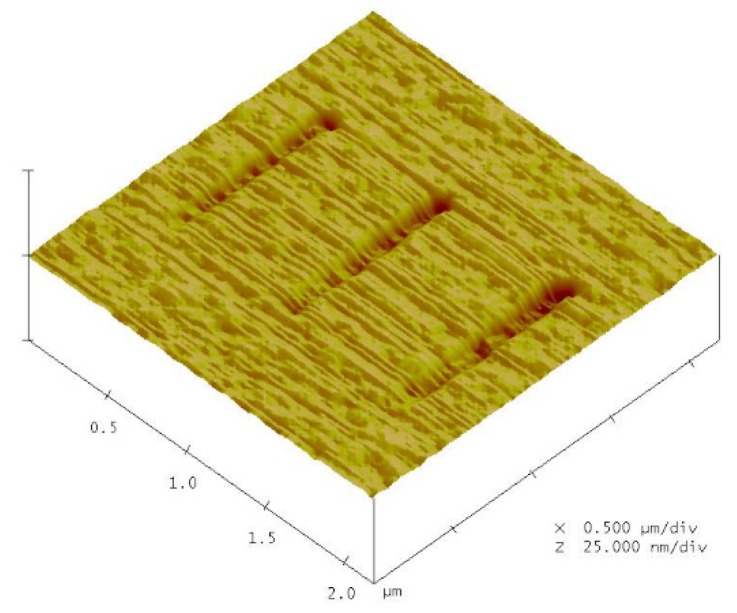
The AFM images for nano-oxidation with probe speed of 0.1 μm·s^−1^ and applied voltage of 9 V.

**Figure 6:**
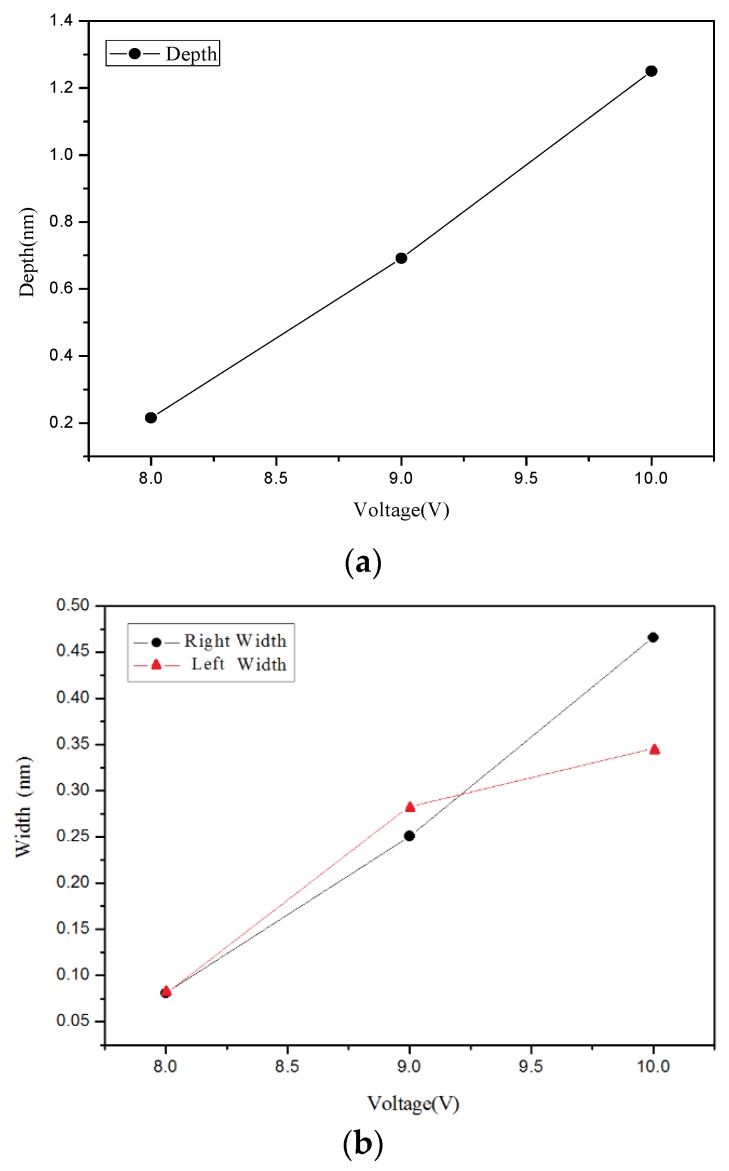
Chart of relationship between line groove depth and width at different applied voltages: (**a**) depth, and (**b**) right width and left width.

**Figure 7:**
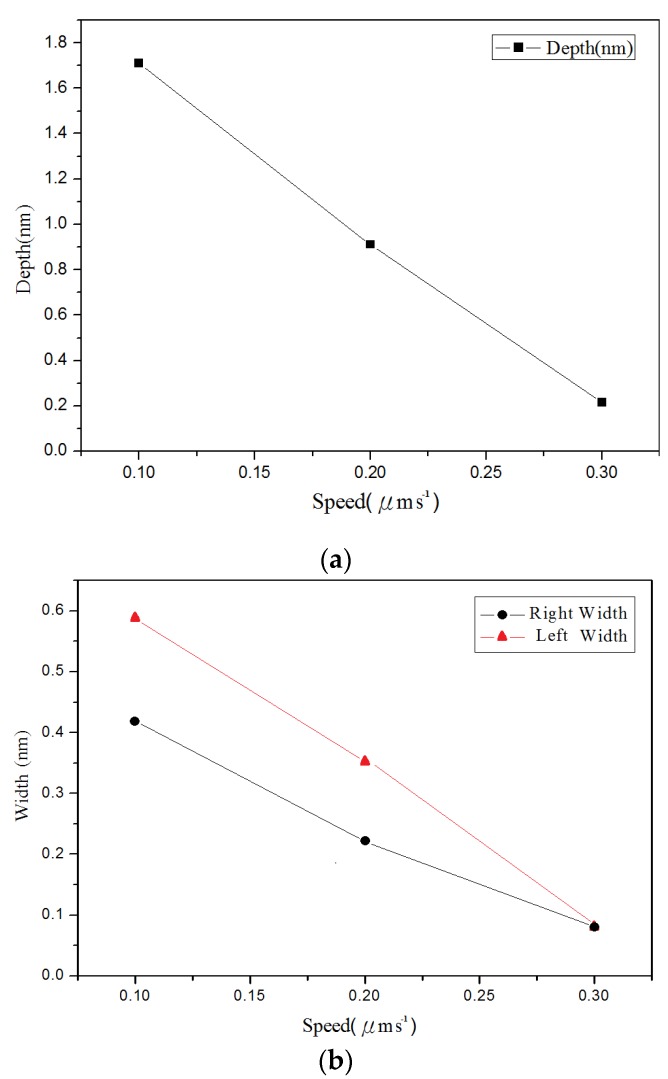
Relationship between the line groove depths and widths in different probe speeds: (**a**) depth, and (**b**) right width and left width.

The results of AFM measurements are presented in Figure 5. Figure 6 and Figure 7 show the changes in the groove depth and width due to the different applied voltages and probe speeds. Figure 5 and Figure 6 show that the groove depth and width increased proportionally with applied voltage. From the Figure 7, it can be found that the influence of probe speeds on the depth and width were the groove depth value decreased with increasing probe speed.

### 4.3. The Performance of Different Prediction Model

**Figure 8:**
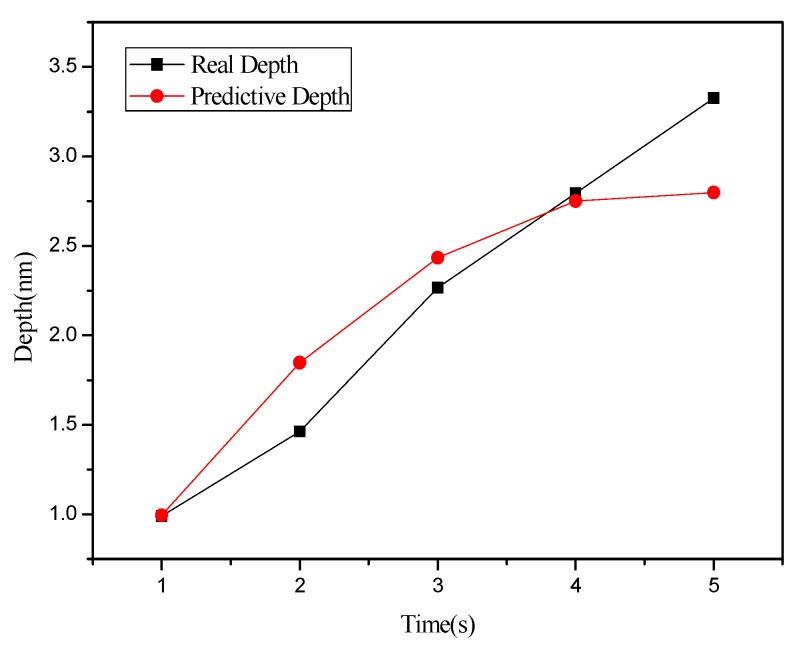
Relationship between depth and oxidation time of probe acquired after point machining by the nano-oxidation technique at a voltage of 8 V.

**Figure 9:**
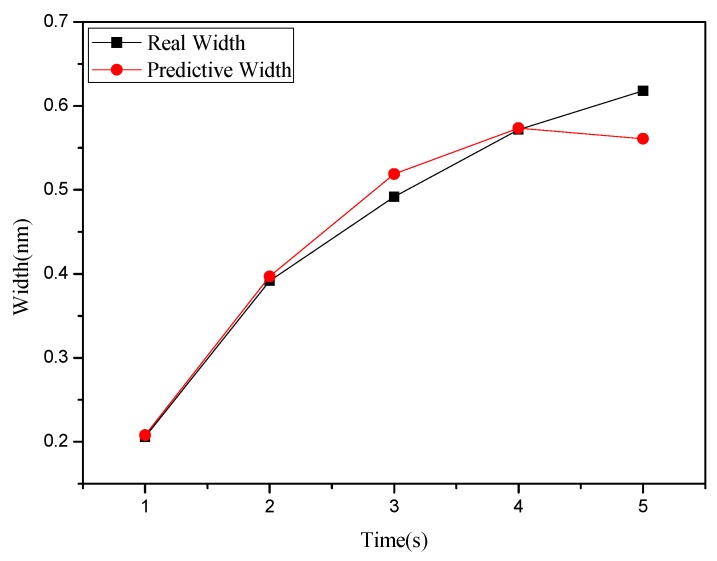
Relationship between left width and oxidation time of probe acquired after point machining by the nano-oxidation technique under a voltage of 8 V.

This study used MRA to explore how the depth and width (left and right side) of the oxide are related to the independent variables set in Table 1 and Table 2. Meanwhile, the predicted values obtained from regression analysis are compared with actual values. Figure 8 shows the relationship between depth and oxidation time of the probe acquired after point machining under the condition of a voltage at 8 V in the nano-oxidation process. The figure shows that the actual values and predicted values differ only when the oxidation time of the probe is 5 s, and the difference is very small. Furthermore, the predicted values in Figure 8 take the optimal Database 5 in Table 1 as independent variables. The calculations showed that the regression model had a judgment coefficient of 94.76%, implying that the results of regression analysis are significant. Figure 9 shows the relationship between left side width and oxidation time of probe acquired after point machining under the condition of voltage at 8 V in the nano-oxidation process. The values in Figure 9 were predicted by using the optimal Database 5 in Table 1 as independent variables. After calculation, it is known that the coefficient of judgment of its regression model is 91.52%. Under the 10 V condition, the data in the optimal Database 4 in Table 1 are used as independent variables. Calculations of the relationship between the right side width and the oxidation time of the probe reveal that the coefficient of judgment of its regression model is 36.43%, implying that the significance level is not high. The calculations indicated that the MRA is highly accurate for predicting depth, but not width. Statistical analyses of the point machining prediction results obtained by MRA for oxide show that the average error rates of depth, left width and right width are 10.15%, 11.66%, and 31.34%, respectively.

**Figure 10:**
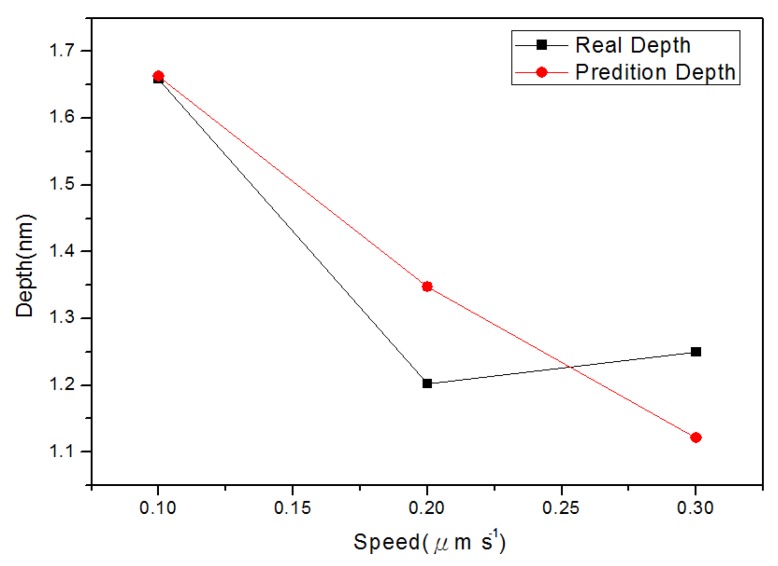
Relationship between depth and probe speed acquired after line machining by the nano-oxidation technique under a voltage of 10 V.

**Figure 11:**
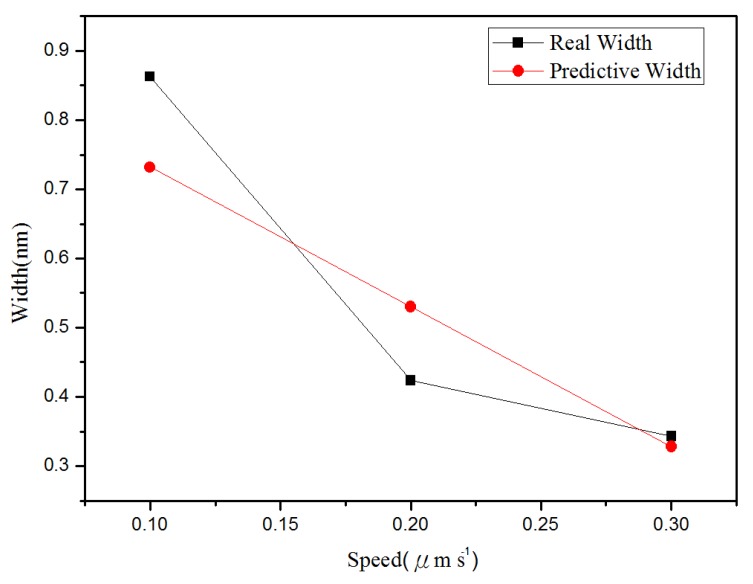
Relationship between left width and probe speed acquired after line machining by the nano-oxidation technique under a voltage of 10 V.

Figure 10 shows the relationship between depth and probe speed acquired after line machining under the condition of voltage of 10 V in the nano-oxidation process. The Figure 10 shows that, when the probe speed increases, the difference between actual values and predicted values increases. The predicted values in Figure 10 are obtained when the best values in Database 5 (Table 2) are used as the independent variables. For the regression model, the coefficient of judgment is 89.05%. Figure 11 shows the relationship between left side width and probe speed acquired after line machining under the condition of voltage at 10 V in the nano-oxidation process. The figure shows that when probe speed increases, the error increases. The predicted values in Figure 11 are obtained when the best values in Database 1 (Table 2) are used as independent variables. After calculation, it is known that the coefficient of judgment of its regression model is 76.33%. Furthermore, when voltage is 8 V, Database 3 in Table 1 is taken as the independent variables. Calculations for the relationship between the right side width and probe speed revealed that the coefficient of judgment of its regression model is 63.28%. When using MRA to predict line machining results for oxide, the average error rates for depth, left width, and right width are 11.04%, 19.84%, and 16.19%, respectively.

**Figure 12:**
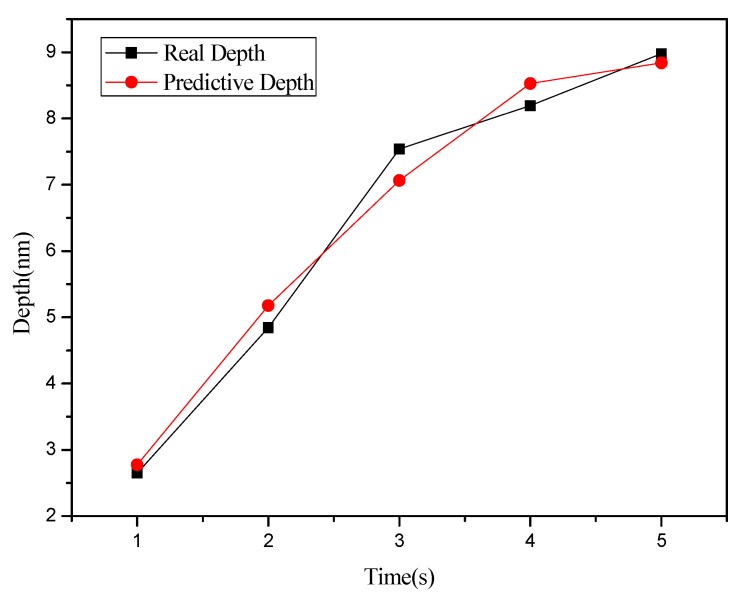
Relationship between depth and probe oxidation time acquired after point machining by the nano-oxidation technique under a voltage of 10 V.

**Figure 13:**
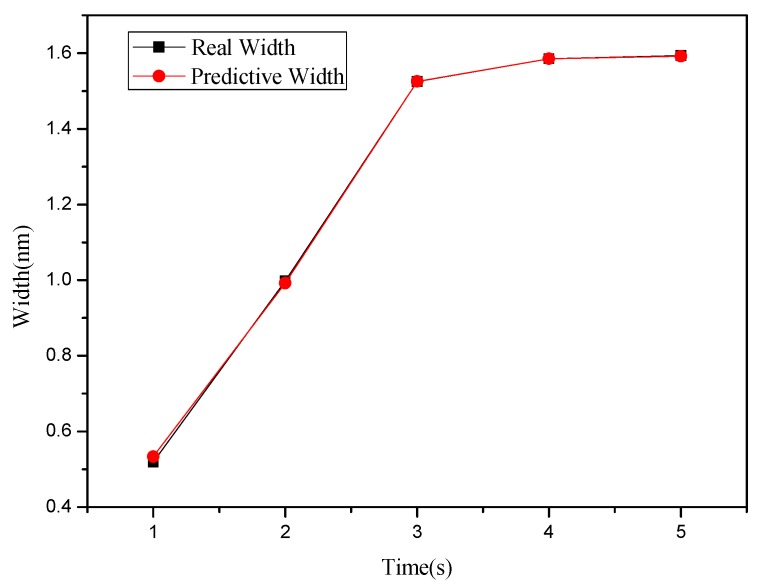
Relationship between left width and oxidation time of probe acquired after point machining by the nano-oxidation technique under a voltage of 10 V.

**Figure 14:**
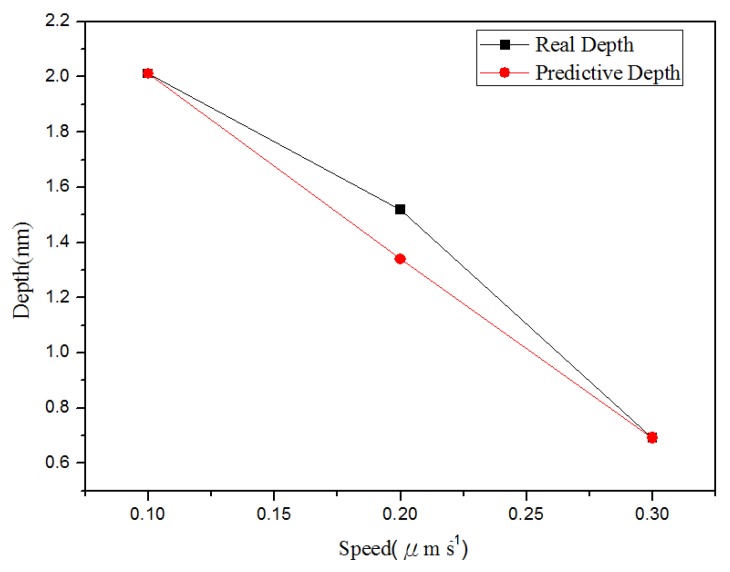
Relationship between depth and probe speed acquired after line machining by the nano-oxidation technique under a voltage of 9 V.

**Figure 15:**
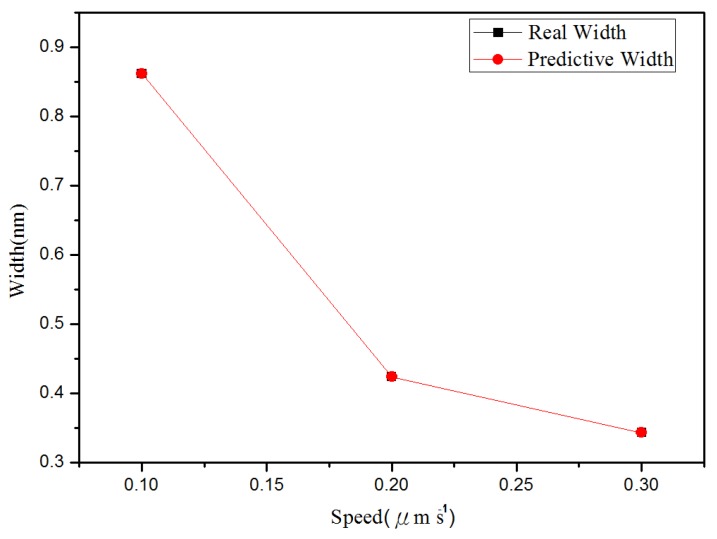
Relationship between left side width and probe speed acquired after line machining by the nano-oxidation technique under a voltage of 10 V.

The BPN algorithm was also used to explore how depth and width (left and right sides) of oxide are related to the independent variables set in Table 1 and Table 2. Meanwhile, the predicted values obtained from BPN analysis are compared with actual values. Figure 12 shows the relationship between depth and oxidation time of the probe acquired after point machining under the condition of voltage at 10 V in nano-oxidation process. The figure shows that the difference between the actual and predicted values is obviously smaller than that in Figure 8, which implies that, compared to MRA, BNP is superior for predicting the depth acquired after point machining. Figure 13 shows the relationship between left width and oxidation time of the probe acquired after point machining under the condition of voltage at 10 V in the nano-oxidation process. The figure shows that the actual and predicted values almost overlap, which implies that BNP is highly accurate for predicting the left width acquired after point machining. The calculations and statistical analyses revealed that, when using BPN to predict point machining results of oxide, the average error rates in depth, left width, and right width are 7.67%, 3.3%, and 11.57%, respectively. Figure 14 shows the relationship between depth and probe speed acquired after line machining under the condition of voltage at 9 V in the nano-oxidation process. The figure shows that the difference between actual values and predicted values is much smaller than that in Figure 10, which implies that the BNP is superior to MRA in terms of predicting the depth acquired after line machining. Figure 15 shows the relationship between left width and oxidation time of probe acquired after point machining under the condition of voltage at 10 V in nano-oxidation process. The actual values and predicted values are highly consistent, which implies the BNP is highly accurate in predicting the left width acquired after point machining. After calculation and statistics are made, the line machining prediction results of oxide by BPN are shown that the average error rates of depth, left width, and right width are 3.69%, 0.22%, and 4.98%, respectively. This implies that BNP is superior to MRA in terms of predicting the depth, left width, and right width after point machining and line machining.

**Figure 16:**
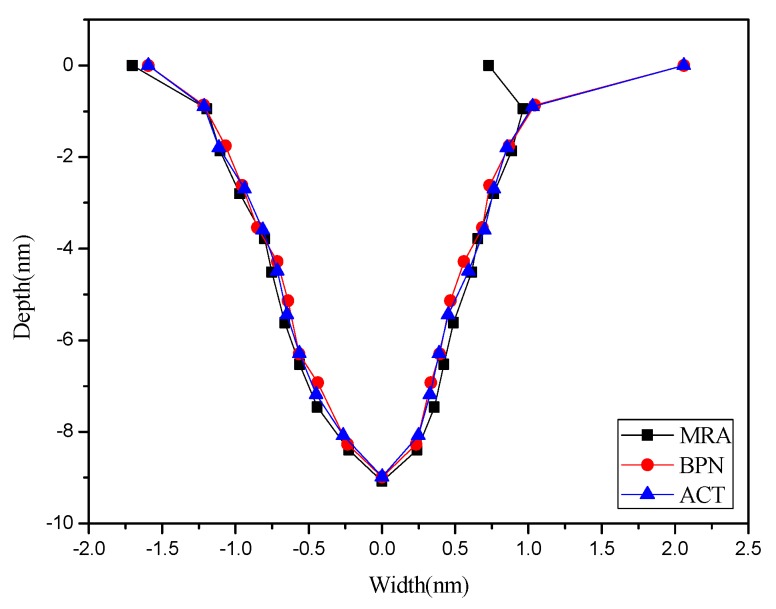
Actual morphology acquired after point machining by the nano-oxidation technique and the morphology of two prediction models when voltage is 10 V and probe oxidation time of 5 s.

**Figure 17:**
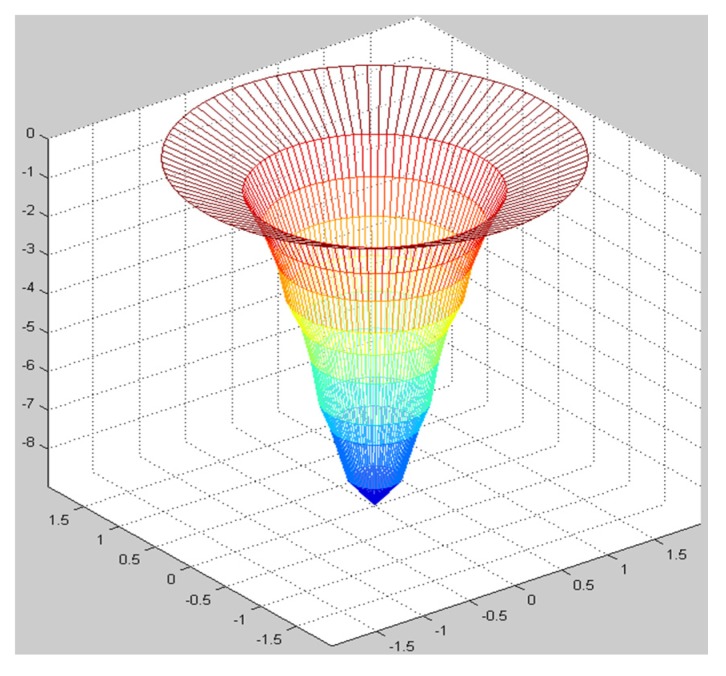
Morphology prediction model acquired after point machining by the nano-oxidation technique when voltage is 10 V and a probe oxidation time of 5 s.

Table 3 shows the mean absolute percentage errors (MAPEs) of two prediction models after point machining and line machining by the nano-oxidation technique. Table 3 shows that the MAPE of depth, left width, and right width predicted by BPN after point machining are 8.02%, 9.68%, and 7.34%, respectively. Additionally, the MAPE values for depth, left width, and right width predicted by BPN after line machining are 4.96%, 8.09%, and 6.77%, respectively. This implies that, when the BPN model is used to predict point machining and line machining, the prediction of depth or width is both better than the MRA model. Therefore, the BPN model was used to visualize the predictions of surface morphology. The prediction results were used to construct a curve, which was then compared and verified with the cross-sectional curve actually measured by AFM. Finally, a visualizable 3D prediction model diagram is established. Figure 8 shows that the actual morphology acquired after point machining by the nano-oxidation technique and the morphology using two prediction models when voltage is 10 V and oxidation time of probe is 5 s. As Figure 16 shows, the error rate between the morphology of point machining predicted by the BPN and the actual morphology is smaller, but the error rate between the morphology of point machining predicted by MRA and the actual morphology is much larger. Figure 17 shows the morphology prediction model acquired after point machining by the nano-oxidation technique under a voltage of 10 V and a probe oxidation time of 5 s.

**Table 3:** MAPEs of two prediction models after point machining and line machining by the nano-oxidation technique.

	Machined Morphology	POINT (%)			LINE (%)		
Prediction Model		Depth	Left	Right	Depth	Left	Right
**MRA**		16.18	20.94	18.96	14.22	18.96	19.6
**BPN**		8.02	9.68	7.34	4.96	8.09	6.77

## 5. Conclusions

This study developed a method of using atomic force microscopy to visualize the morphology of diamond-like carbon (DLC) thin films subjected to the nano-oxidation technique. After establishing a databank for process parameters and feature sizes of thin films, this study used multiple regression analysis (MRA) and a back-propagation neural network (BPN) to perform the algorithm. Experimental results shows that the MAPE values for depth, left width, and right width predicted by BPN after point machining are 8.02%, 9.68%, and 7.34%, respectively. Furthermore, the MAPE of depth, left width, and right width predicted by BPN after line machining are 4.96%, 8.09%, and 6.77% respectively. The predicted data can also be used to construct predictions of the cross-sectional morphology of DLC thin film after treatment by the nano-oxidation process. The proposed prediction models can also predict the complete 3D morphology after processing, rather than just get the height or width of the data. Therefore, experimental results of this study are applicable in the DLC film surface machining process, and the complex structure pattern of processing applications. Further research may provide a highly-reliable DLC nanostructure fabrication technology that can be used for nanostructure mold production.
